# Stroking stimulation of the skin elicits 50-kHz ultrasonic vocalizations in young adult rats

**DOI:** 10.1186/s12576-020-00770-1

**Published:** 2020-09-16

**Authors:** Rie Shimoju, Hideshi Shibata, Miyo Hori, Mieko Kurosawa

**Affiliations:** 1 Center for Medical Sciences, International University of Health and Welfare, 2600-1 Kitakanemaru, Otawara, Tochigi 324-8501 Japan; 2 Laboratory of Veterinary Anatomy, Institute of Agriculture, Tokyo University of Agriculture and Technology, 3-5-8 Saiwai‐cho, Fuchu, Tokyo 183-8509 Japan; 3 Foundation for Advancement of International Science, 3-24-16 Kasuga, Tsukuba, Ibaraki 305-0821 Japan; 4 Department of Pharmaceutical Sciences, International University of Health and Welfare, 2600-1 Kitakanemaru, Otawara, Tochigi 324-8501 Japan

**Keywords:** Stroking stimulation, Ultrasonic vocalization, 50-kHz calls, Call subtype, Positive emotion, Rat

## Abstract

The present study aimed to clarify if stroking stimulation of the skin produces positive emotion in rats. 50-kHz ultrasonic vocalizations (USVs) were recorded as an index of the positive emotion. Stroking stimulation was applied to the ventral, dorsal, or head region of the body while the rat was in a vertical holding condition. Rats emit abundant 50-kHz USVs in response to stroking, and the number of the USVs was not different among these three stimulated regions. Other stimulations, such as light touching of the abdominal area, swinging of the body back and forth, or stroking of the external genitalia under vertical holding condition, produced significantly less 50-kHz USVs. Furthermore, different call subtypes were observed during and after stroking of the ventral region. In particular, “Trill” calls, a representative index of positive emotion, were dominant after stimulation. These results suggest that stroking of the skin induces positive emotional states.

## Introduction

Sensory stimulation of the skin alters physiological functions via motor and autonomic nerves. We have reported that somatic stimulation reflexively changes various visceral functions in anesthetized animals. The responses were dependent on the stimulus modalities [1–3]. For example, arterial pressure, heart rate, and adrenal catecholamine secretion increase in response to noxious mechanical stimulation (pinching) [1, 2], whereas they decrease in response to innocuous mechanical stimulation (stroking) of the skin in anesthetized animals [1, 4]. These decreases in the arterial pressure and heart rate are also observed in conscious animals [5], and the duration of the responses are much longer than those in anesthetized animals. These results suggest that the contribution of emotion can be important. In accordance, stroking stimulation is demonstrated to have anxiolytic effects in conscious animals [6]. Furthermore, we have shown that stroking stimulation increases dopamine release in the nucleus accumbens [7, 8], which contributes to the occurrence of the positive emotion. However, it is not clear whether stroking stimulation can alter emotional states in rats.

Juvenile and adult rats emit ultrasonic vocalizations (USVs) that can be classified into two basic types by peak frequency and call duration [9, 10]. One type is the “22-kHz” USV (frequency, 20–30 kHz; call duration, 300 to over 3000 ms), which is observed in negative aversive states, such as predator exposure, social defeat, and foot shock [11–14]. The other type is the “50-kHz” USV (35–80 kHz, 10–150 ms), which has been proposed to reflect a positive state akin to human joy and laughter. Such calls are associated with positive affective contexts, including mating, the availability of food, anticipation of positive rewards, administration of psychostimulant drugs, and rough-and-tumble play [9, 15–17].

A significant negative correlation has been demonstrated between the 50-kHz vocalization rate and the approach latency time, an index of positive behavior [18]. Thus, the number of 50-kHz USVs was employed in many USV studies for evaluating the intensity of positive emotion [19]. Furthermore, 50-kHz USVs are divided generally into two main types of calls, monotonous “Flat” and “Frequency-modulated (FM)” calls [20]. The 50-kHz USVs were further divided into as many as 14 different subtypes by Wright et al. [21]. Recently, accumulating evidence demonstrated that the variety of acoustic features are susceptible to experimental manipulation and may contain important information not encoded by the absolute number of calls.

In the present study, we examined the effect of stroking stimulation on both the absolute number of USVs and the call subtypes demonstrated by Wright et al. [21]. We applied stroking stimulation to the ventral region, including the chest, abdomen, pelvis, and thigh, under vertical holding condition since the stimulation under vertical holding condition has been shown to produce anxiolytic and hypotensive responses [5, 6]. We also applied stroking stimulation to other cutaneous regions (dorsal and head regions) to elucidate the stimulus site specificity for evoking USVs. In addition, stroking of the external genitalia, light touching of the abdominal area, and swinging the body back and forth were performed under vertical holding condition to clarify the stimulus modality specificity for evoking USVs.

## Materials and methods

### Animals

The experiments were performed on 18 male Wistar/ST rats (7–9 weeks old, 260–310 g). The animals were maintained under temperature-controlled conditions (23 ± 1 °C) with a 12-h light/dark cycle (Showa Co. Ltd., Tokyo). Commercial rodent chow (Labo-MR stock, Nosan Corporation, Kanagawa) and tap water were provided ad libitum, except during experiments. All experiments were performed in accordance with the Japanese Physiological Society’s Guide for the Care and Use of Laboratory Animals. The study protocol was approved by the animal ethics committee of the International University of Health and Welfare.

The animals were housed individually for 2 weeks and received 2 min of stroking stimulation (the ventral region) once a day beginning at 1 week prior to the start of the experiments in order to acclimate the rats to the stimulation. Furthermore, for 2 consecutive days immediately before the experiments, each rat was placed in a standard polycarbonate home cage (W27 cm × L44 cm × H18 cm) and left undisturbed for at least 30 min. These acclimation/habituation procedures were intended to extinguish the animals’ anxiety. All manipulations were conducted by the same experimenter.

### Recordings of USVs

The experiments were conducted between 09:00 to 16:00 h. USVs of each rat were recorded individually in a home cage, and recordings of USVs were performed by an UltraSoundGate 116H audio device with a CM16/CMA microphone (Avisoft Bioacoustics, Berlin, Germany). The ultrasonic microphone was mounted centrally at approximately 15 cm distance from the rats. The sampling rate was 300 kHz with 16-bit resolution. During all recordings, the intensity gain remained at the same level for all rats. For acoustical analysis, recordings were transferred to SASLab Pro (version 4.2, Avisoft Bioacoustics). Spectrograms were generated with a fast Fourier transform (FFT)-length of 512 points and an overlap of 75% (FlatTop window, 100% frame size). Each call was visually and acoustically identified by a trained observer.

### Experimental design

The present study consisted of three experiments (experiment I, II, and III). In experiment I, responses of the USVs to stroking stimulation of the ventral region, including the chest, abdomen, pelvis, and thigh, were analyzed by both the number and acoustic features of USVs in 6 rats. USVs were recorded for 6 consecutive minutes (pre-stimulation for 1 min, during stimulation for 1 min, and post-stimulation for 4 min). The 50-kHz calls were categorized during both the 60-s stimulus period and the 60-s post-stimulus period, according to the modified 14-subtype scheme described by Wright et al. [21].

In experiment II, another cohort of rats (*n* = 6) were used in order to determine the stimulus modality specificity for evoking 50-kHz USVs. Therefore, the responses of 50-kHz USVs to stroking of the ventral region were compared with the responses to other stimulations, including stroking stimulation of the external genitalia area, light touching of the ventral region, and swinging the body back and forth. Each of the other stimulations was delivered on separate days. Light touching of the ventral region was delivered on the first day, stroking of the external genitalia area was delivered on the second day, swinging the body back and forth was delivered on the third day, and the sham stimulation (vertical holding without stroking) was delivered on the fourth day. Stroking of the ventral region was performed each day, and the effects of stroking the ventral region on the 50-kHz USVs were compared to those observed during each of the other stimulations. All stimulations were delivered for 30 s, and the USVs were recorded during the 30-s stimulus period.

In experiment III, another cohort of rats (*n* = 6) were used in order to determine the stimulus site specificity for evoking 50-kHz USVs. Stroking stimulation was applied to three different cutaneous areas (ventral, dorsal part of the body, and head regions), and USVs were recorded during the 30-s stimulus period.

### Stimulations

**Fig. 1:**
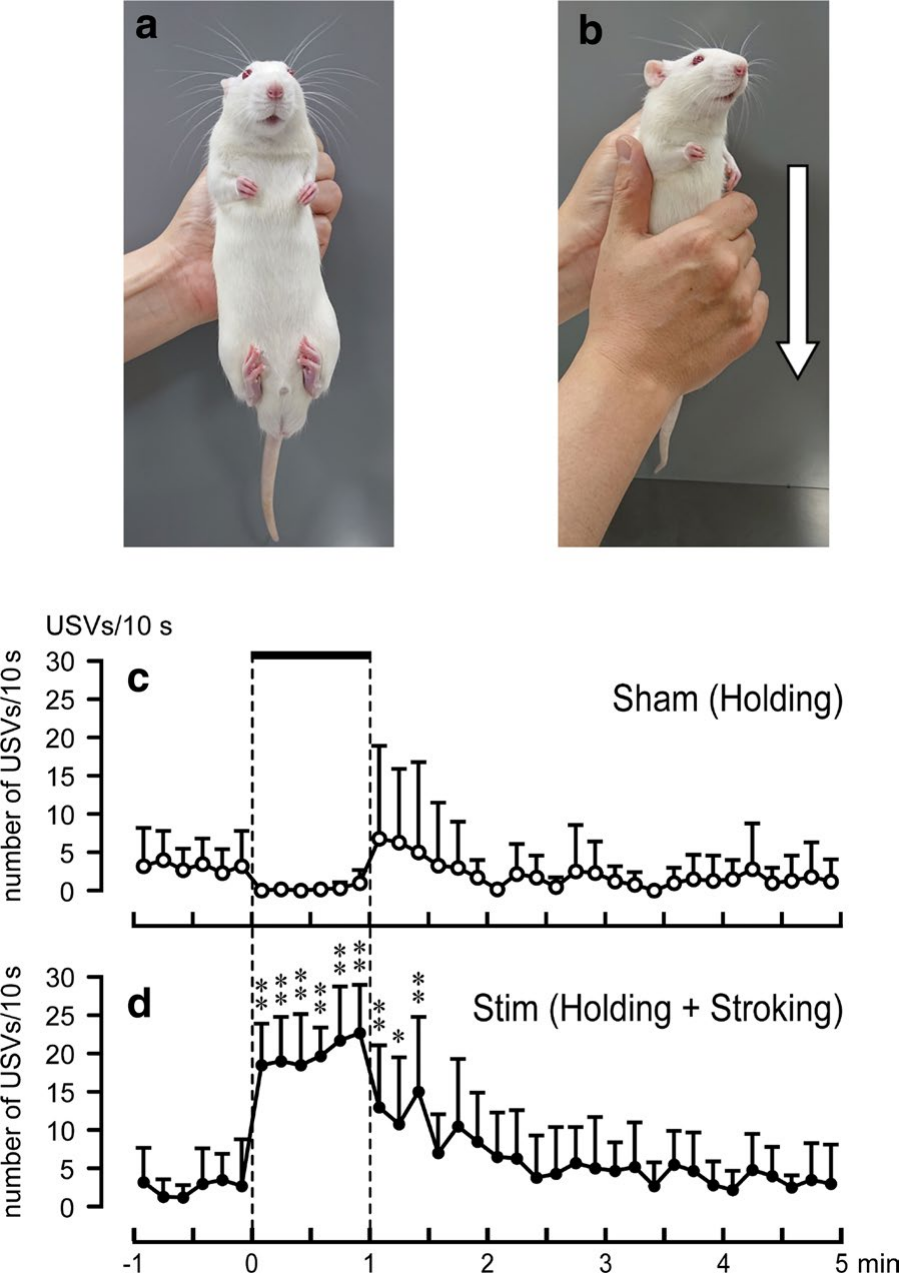
Method of stroking stimulation under vertical holding condition. **a** Vertical holding. The experimenter gently squeezed the rat’s upper back skin, pulling backward without stroking (sham stimulation). **b** Stroking of the ventral region. The stimulation was applied towards the lower part from the upper part of the body (as indicated by the arrow) with the right hand at a frequency of approximately 1 Hz. **c**, **d** Responses of 50-kHz USVs to sham (**c**) or stroking (**d**) stimulation. Ordinates, number of 50-kHz USVs per 10 s; abscissa, 0 indicates the onset of stimulation. Horizontal bar indicates the 60-s stimulus or sham stimulus period. *p < 0.05, **p < 0.01, vs pre-stimulus control value, using ANOVA followed by Dunnett’s multiple comparison test. *n* = 6

All stimulations were applied under vertical holding condition, in which the experimenter hung a rat perpendicularly and gently squeezed the upper back skin by pulling its skin backwards with the left hand. The appropriate status of the vertical holding condition was confirmed by observing that the rat flexes and adducts its hindlimbs and forelimbs (Fig. 1a). Vertical holding alone was referred to as sham experiments in the present study.

In experiment I, stroking stimulation of the ventral region (chest, abdomen, pelvis, and thigh) was applied towards the lower part from the upper part of the body [5, 8] for 60 s at a frequency of approximately 1 Hz with the experimenter’s right hand (Fig. 1b). The frequency of stimulation was confirmed using a metronome without sound.

In experiment II, stroking stimulation of the external genitalia (area around the urethral opening) was applied for 30 s. Light touching of the abdomen was conducted by gently touching on the abdominal region with the experimenter’s four fingers (without the thumb) at approximately 1 Hz for 30 s. Swinging of the body back and forth was conducted at approximately 1 Hz for 30 s.

In experiment III, stroking stimulation of the dorsal region (back and rump) was applied beginning at the upper part of the body and moving towards the lower part, and stimulation of the head region was applied from the forehead towards the occipital region and nape of the neck for 30 s, at a frequency of approximately 1 Hz.

### Statistics

All values were expressed as mean ± SD and were analyzed using commercial software (IBM SPSS Statistics 23; International Business Machines Co, Armonk, NY, USA). Paired *t*-test was employed for the following comparisons: the differences in the pre-stimulus basal number (mean value for 60 s before stimulation) of 50-kHz USVs between sham and ventral stroking groups, differences in the number of each call subtype between the 60-s stimulus period and the 60-s post-stimulus period, differences in the number of 50-kHz USVs between ventral stroking and the other stimulation groups, and differences in the number of 50-kHz USVs among ventral, dorsal, and head stroking groups. Changes over time within a group (sham or ventral stroking group) were analyzed by repeated-measures one-way ANOVA followed by Dunnett’s test. The statistical analysis of the acoustic features (peak frequency, call duration, and peak amplitude) among representative call subtypes was performed by repeated-measures ANOVA followed by post hoc Tukey’s HSD test. Probability values of less than 5% were considered significant.

## Results

The pre-stimulus basal number of 50-kHz USVs in the sham (holding) group (2–4 calls/10 s, Fig. 1c) was not significantly different from that of the stroking group (1–4 calls/10 s, Fig. 1d). Stroking of the ventral region produced rapid and significant increases in 50-kHz USVs (*p* < 0.001), and the increases persisted (19–23 calls/10 s) during the 60-s stimulus period (Fig. 1d). After cessation of stimulation, the number of 50-kHz USVs gradually decreased and returned to the pre-stimulus levels at 30–40 s after cessation of the stimulation. The USVs in the sham group tended to decrease during 60-s holding period, but the decreases were not significant (Fig. 1 c). The total number of 50-kHz USVs during the 60-s stimulus period in the stroking group was 120 ± 31 calls and the value was significantly (*p* < 0.001) larger than that of the sham group (2 ± 2 calls).

**Fig. 2:**
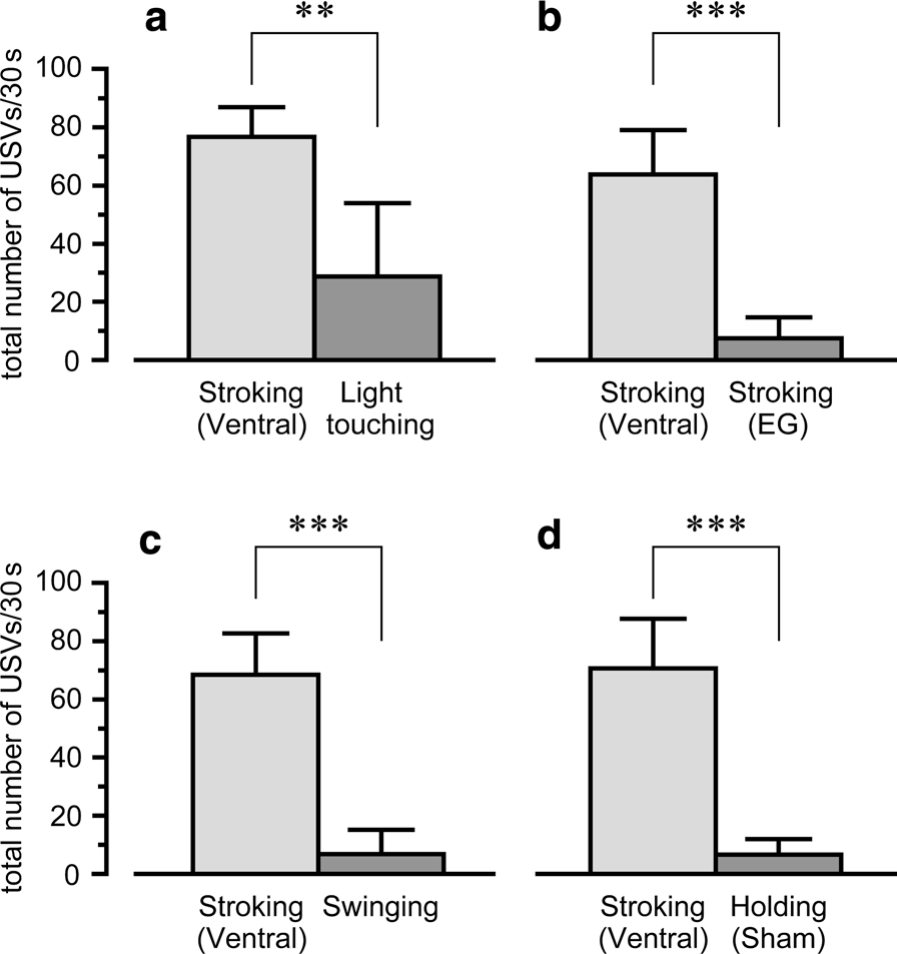
Responses of 50-kHz USVs to various stimulation. The number of 50-kHz USVs during ventral stroking (light gray column) was compared with that of other stimulations (dark gray column): light touching (**a**), stroking of the external genitalia (EG) (**b**), and swinging of the body (**c**). A comparison was also made between ventral stroking and holding without stroking (sham) (**d**). Ordinates, total number of 50-kHz USVs during 30-s stimulus period. **p < 0.01 ***p < 0.001, by paired *t*-test. *n* = 6

The responses of 50-kHz USVs to stroking the ventral region was compared with that of other stimulations (light touching the abdominal area, stroking the external genitalia area, and swinging the body back and forth) in another 6 rats during a 30-s stimulus period. The number of 50-kHz USVs during light touching (29 ± 25 calls) was significantly less than that of the ventral stroking (77 ± 10, *p* < 0.01). Similarly, the numbers of 50-kHz USVs during stroking the external genitalia (8 ± 7 calls) and swinging the body (7 ± 8 calls) were significantly smaller than those of respective controls (stroking the ventral region) (*p* < 0.001) (Fig. 2). The number of 50-kHz USVs elicited during ventral stroking in each stimulus pattern was not statistically different (light gray columns in Fig. 2a–d).

The number of 50-kHz USVs in the sham group (holding without stimulation) was confirmed to be significantly less (7 ± 5 calls, *p* < 0.001) than that in the ventral stroking group (Fig. 2d).

The effects of stroking the three different skin regions (ventral, dorsal, and head) on 50-kHz USVs under vertical holding condition were compared in 6 animals. There were no significant differences in total number of 50-kHz USVs (ventral, 75 ± 16 calls; dorsal, 81 ± 24 calls; and head, 86 ± 18 calls) during the 30-s stimulus period.

**Fig. 3:**
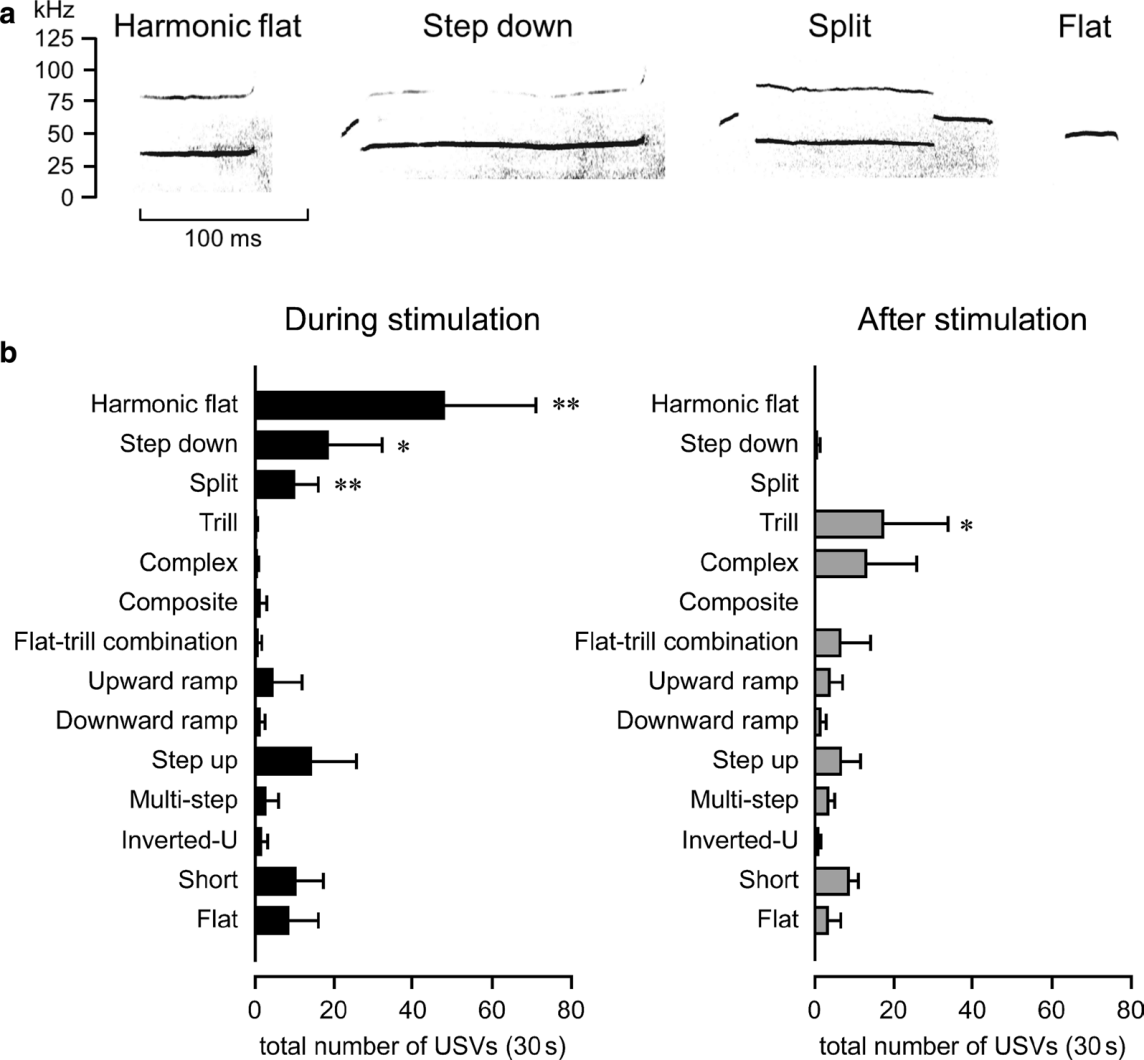
Subtypes of 50-kHz USVs evoked by stroking of the ventral region. **a** Representative sonograms during stroking stimulation. **b** Absolute number of call subtypes during the 60-s stimulus period (left panel) and the 60-s post-stimulus period (right panel). The sonograms were obtained from four different rats. The data are based on 719 calls during the 60-s stimulus period and 385 calls during the 60-s post-stimulus period (*n* = 6). *p < 0.05, **p < 0.01, between groups, using paired *t*-test

Subtypes of 50-kHz USVs evoked by stroking stimulation of the ventral region were investigated in the same cohort of rats shown in Fig. 1 (n = 6). Various 50-kHz subtypes were observed during both the 60-s stimulus period and 60-s post-stimulus period. Three representative examples of the call subtypes (“Harmonic flat”, “Step down”, and “Split”) evoked during the stimulus period are shown in Fig. 3a. In addition, the monotonous “Flat” call is also shown in Fig. 3a for comparison with the “Harmonic flat” call. In the present study, 22-kHz calls were not observed.

The peak frequency, call duration, and peak amplitude of these representative calls are summarized in Table 1. Mean peak frequency was approximately 40–50 kHz in each call subtype, and there were no significant differences in the mean peak frequency among the subtypes. On the other hand, the mean call duration of “Flat” (non-harmonic flat) call was significantly shorter than that of “Step down” (*p* < 0.01) and “Split” calls (*p *< 0.01). Mean amplitude of “Harmonic flat” was significantly larger than other subtypes (Step down, 40.9 ± 20.4%; Split, 42.2 ± 25.9%; Flat, 27.9 ± 14.0%, expressed as percentage of the “Harmonic flat” amplitude).

**Table 1:** Call parameters (mean peak frequency, mean call duration and mean peak amplitude) of representative call subtypes during stroking

	Harmonic flat	Stepdown	Split	Flat
Mean peak frequency (kHz)	39.5 ± 7.3	41.2 ± 7.1	39.9 ± 7	48.5 ± 6.4
Mean call duration (ms)	117.6 ± 56.7	176.6 ± 71.1**	178.5 ± 38.4**	43.8 ± 17.2
Mean peak amplitude (dB)	− 30.4 ± 7.4	− 35.7 ± 10.2	− 38.6 ± 9.7	− 42.2 ± 3.3
Mean peak amplitude (%)	100	40.9 ± 20.4^##^	42.2 ± 25.9^##^	27.9 ± 14.0^##^

**p < 0.01 vs “Flat” call, ^##^*p* < 0.01 vs “Harmonic flat” call

The absolute number of each call subtype during both the 60-s stimulus period and the 60-s post-stimulus period was investigated (Fig. 3b). During the stimulus period, 48 ± 23 calls were “Harmonic flat”, but “Harmonic flat” calls were not observed after stimulation. “Step down” (18 ± 14 calls) and “Split” (10 ± 6 calls) calls were also observed during stimulus period, but “Step down” calls were infrequent (1 ± 0 calls) and “Split” (0 ± 0 calls) calls did not occur after stimulation. In contrast, “Trill” (17 ± 16 calls) calls appeared after stimulation, and were rarely observed during the stimulus period.

## Discussion

Results of the present study showed that young adult rats emit 50-kHz USVs in response to stroking stimulation of the ventral region under vertical holding condition. Furthermore, it was demonstrated that the 50-kHz USVs include various subtypes, and that the composition of the subtypes was different between during and after stimulation.

Since the rate of 50-kHz USVs is shown to relate to the degree of positive reinforcement [19], we first investigated the effects of stroking stimulation on the rate of 50-kHz USVs. The present study demonstrated that young adult rats emit abundant 50-kHz USVs in response to stroking stimulation of the ventral region under vertical holding condition. Fifty-kHz USVs were also observed by stroking other cutaneous regions (dorsal or head regions), and there were no differences in the number of calls among the stimulated sites (ventral, dorsal, and head). These results suggest that stroking of the skin under vertical holding condition produces positive emotion irrespective of the cutaneous stimulus sites, at least in the rats acclimatized to the stimulation for 1 week.

We attempted to elucidate factors eliciting USVs to stroking stimulation in the present study. First, we examined a possibility that the 50-kHz USVs evoked by stroking of the ventral region is due to sexual arousal following the stimulation of the lower abdomen including the external genitalia, stroking stimulation around the external genitalia area was applied. The number of USVs evoked by stimulation of the external genitalia area was significantly less than that of ventral stroking (Fig. 2). These results demonstrate that 50-kHz USVs emitted by stroking of the ventral region were not due to sexual arousal. Second, we examined a possibility that vestibular sensation contributes to the 50-kHz USVs evoked by ventral stroking, since the body of the rats swung back and force during stroking of the ventral (or dorsal) region. However, swinging the body alone produced few 50-kHz USVs. Therefore, the evidence suggests that excitement of the cutaneous receptors is important to elicit the 50-kHz USVs.

Stroking stimulation (tactile stimulation with movement) over the hairy skin area activates various mechanoreceptors, such as hair follicle receptors, Merkel discs, and Ruffini corpuscles. In addition, warm receptors may also respond to the experimenter’s bare hand in the present study. The fact that light touching on the same skin area (tactile stimulation without movement) induced few 50-kHz USVs (Fig. 2) indicates that the contribution of receptors responding to the tactile movement is important for inducing 50-kHz USVs. It is noteworthy that C-tactile fibers respond particularly well to cutaneous stroking stimulation, but poorly to rapid skin deformation such as light touching stimulation [22, 23]. Since C-tactile afferent fibers constitute a peripheral pathway for pleasant tactile stimulation [24], it is suggested that tactile C-afferent fibers may be important for producing 50-kHz USVs in response to tactile stimulation. Further investigation on the afferent mechanisms is required.

In the present study, the 50-kHz USVs were classified into several call subtypes. During stroking stimulation 13 out of the 14 subtypes (except “Trill with jumps”) proposed by Wright et al. [21] were observed. One new call subtype was identified that was not described in the Wright et al.’s scheme [21]. The new subtype was monotonous call with harmonics, and was defined as “Harmonic flat”. It was shown that 22-kHz (20–30 kHz) USVs occasionally appeared with 40 and 60-kHz USVs, and this is thought to be noise generated by reverberations from high decibel 22-kHz USVs [25]. Since the amplitude of the “Harmonic flat” call was larger than that of other subtype calls, there is a possibility that the harmonics were caused by reverberations. However, the fundamental frequency of the “Harmonic flat” call (39.5 ± 7.3 kHz) is higher than that of 22-kHz USVs and the call duration of the “Harmonic flat” call (117.6 ± 56.7 ms) is shorter than that of 22-kHz USVs (300–3000 ms [9]), suggesting that the “Harmonic flat calls” were not in the group of 22-kHz USVs.

Although the “Harmonic flat” calls might be elicited due to acoustic byproduct, these calls may appear in other situations than stimulation of rats in the vertical position. For example, the administration of amphetamine, a psychotropic agent, increases call amplitude, suggesting that the large amplitude of the present “Harmonic flat” calls is due to some emotional changes. In addition, sonograms similar to the “Harmonic flat” calls have been reported during copulation [14, 26], carbachol injections into the nucleus accumbens [27], and rat pup calls during maternal separation [28]. In any case, further research is needed to clarify whether the “Harmonic flat” call is related to emotion or only byproducts of mechanical noise.

We found that “Trill” calls were evoked characteristically during the 60-s post-stimulus period. Abundant “Trill” calls are observed during social play [29], mating [30], and approach to a partner of anticipating play (i.e., its former cage mate) [20]. These results indicate that the rats receiving stroking of the ventral region entered a positive emotional state.

Rats habituated to the present vertical holding method emitted neither 50-kHz USVs nor aversive 22-kHz USVs during vertical holding without stroking. In addition, we did not observe any indices of stress, such as abducted lower limbs and increased abdominal muscle tension, defecation, urination, and audible calls during dorsal immobilization [31, 32].

When a pup is grasped for transport by the mother or the experimenter, the pup characteristically responds by reducing its general activity. Typically, a pup actively flexes and adducts its hindlimbs and forelimbs, which is called the transport response [33]. In the present study, the hindlimbs and forelimbs of the habituated rats also showed a similar posture of the transport response (Fig. 1a). Furthermore, the pups also showed reduced indices of stress during transport response (e.g., reduction in heart rate, distress vocalizations, and induction in parasympathetic activity) [34].

Our previous study showed that tickling, a model of human joy, induces 50-kHz USVs in juvenile rats via dopamine in the nucleus accumbens, a reward center [35]. We also reported that stroking stimulation under vertical holding condition increased accumbal dopamine release in adult rats [8]. Our results suggest that the accumbal dopamine release relates to the emission of 50-kHz USVs to stroking stimulation under vertical holding condition.

## Conclusions

In conclusion, the main finding of our study is that stroking of the cutaneous ventral, dorsal, and head region under vertical holding condition elicits abundant 50-kHz USVs in young adult rats that were acclimatized to the stroking stimulation for 1 week. The number of 50-kHz USVs evoked by stroking of the ventral region was significantly greater than that of other stimulations (light touching, swinging the body back and forth), suggesting that the cutaneous stroking stimulation is important for evoking 50-kHz USVs. Analysis of the subtypes showed that “Trill” call, an index of typical positive emotion, is dominant after stimulation. Our results suggest that stroking of the skin induces positive emotion in rats. The functional meaning of call subtypes evoked during and after stroking stimulation remains to be determined.

## Data Availability

The datasets used and/or analyzed during the current study are available from the corresponding author on reasonable request.

## References

[1] Araki T, Ito K, Kurosawa M, Sato A (1984). Responses of adrenal sympathetic nerve activity and catecholamine secretion to cutaneous stimulation in anesthetized rats. Neuroscience.

[2] Kimura A, Ohsawa H, Sato A, Sato Y (1995). Somatocardiovascular reflexes in anesthetized rats with the central nervous system intact or acutely spinalized at the cervical level. Neurosci Res.

[3] Sato A, Sato Y, Schmidt RF (1997). The impact of somatosensory input on autonomic functions. Rev Physiol Biochem Pharmacol.

[4] Kurosawa M, Lundeberg T, Agren G, Lund I, Uvnäs-Moberg K (1995). Massage-like stroking of the abdomen lowers blood pressure in anesthetized rats: influence of oxytocin. J Auton Nerv Syst.

[5] Lund I, Lundeberg T, Kurosawa M, Uvnas-Moberg K (1999). Sensory stimulation (massage) reduces blood pressure in unanaesthetized rats. J Auton Nerv Syst.

[6] Uvnäs-Moberg K, Alster P, Lund I, Lundeberg T, Kurosawa M, Ahlenius S (1996). Stroking of the abdomen causes decreased locomotor activity in conscious male rats. Physiol Behav.

[7] Maruyama K, Shimoju R, Ohkubo M, Maruyama H, Kurosawa M (2012). Tactile skin stimulation increases dopamine release in the nucleus accumbens in rats. J Physiol Sci.

[8] Shimoju R, Masaoka M, Takaoka K, Shibata H, Kurosawa M (2018). Contribution of opioids to the responses of dopamine release in the nucleus accumbens to tactile stimulation in rats. J Physiol Sci.

[9] Brudzynski SM (2013). Ethotransmission: communication of emotional states through ultrasonic vocalization in rats. Curr Opin Neurobiol.

[10] Wöhr M, Schwarting RK (2013). Affective communication in rodents: ultrasonic vocalizations as a tool for research on emotion and motivation. Cell Tissue Res.

[11] Blanchard RJ, Blanchard DC, Agullana R, Weiss SM (1991). Twenty-two kHz alarm cries to presentation of a predator, by laboratory rats living in visible burrow systems. Physiol Behav.

[12] Cuomo V, Cagiano R, De Salvia MA, Maselli MA, Renna G, Racagni G (1988). Ultrasonic vocalization in response to unavoidable aversive stimuli in rats: effects of benzodiazepines. Life Sci.

[13] Kaltwasser MT (1990). Startle-inducing acoustic stimuli evoke ultrasonic vocalization in the rat. Physiol Behav.

[14] Thomas DA, Barfield RJ (1985). Ultrasonic vocalization of the female rat (*Rattus norvegicus*) during mating. Anim Behav.

[15] Panksepp J, Burgdorf J (2000). 50-kHz chirping (laughter?) in response to conditioned and unconditioned tickle-induced reward in rats: effects of social housing and genetic variables. Behav Brain Res.

[16] Panksepp J, Burgdorf J (2003). “Laughing” rats and the evolutionary antecedents of human joy?. Physiol Behav.

[17] Ahrens AM, Ma ST, Maier EY, Duvauchelle CL, Schallert T (2009). Repeated intravenous amphetamine exposure: rapid and persistent sensitization of 50-kHz ultrasonic trill calls in rats. Behav Brain Res.

[18] Hori M, Hayashi T, Nakagawa Y, Sakamoto S, Urayama O, Murakami K (2009). Positive emotion-specific changes in the gene expression profile of tickled rats. Mol Med Rep.

[19] Burgdorf J, Panksepp J (2001). Tickling induces reward in adolescent rats. Physiol Behav.

[20] Burgdorf J, Kroes RA, Moskal JR, Pfaus JG, Brudzynski SM, Panksepp J (2008). Ultrasonic vocalizations of rats (*Rattus norvegicus*) during mating, play, and aggression: behavioral concomitants, relationship to reward, and self-administration of playback. J Comp Psychol.

[21] Wright JM, Gourdon JC, Clarke PB (2010). Identification of multiple call categories within the rich repertoire of adult rat 50-kHz ultrasonic vocalizations: effects of amphetamine and social context. Psychopharmacology.

[22] Iggo A (1960). Cutaneous mechanoreceptors with afferent C fibres. J Physiol.

[23] Löken LS, Wessberg J, Morrison I, McGlone F, Olausson H (2009). Coding of pleasant touch by unmyelinated afferents in humans. Nat Neurosci.

[24] Pawling R, Cannon PR, McGlone FP, Walker SC (2017). C-tactile afferent stimulating touch carries a positive value. PLoS ONE.

[25] Reno JM, Marker B, Cormack LK, Schallert T, Duvauchelle CL (2013). Automating ultrasonic vocalization analyses: the WAAVES program. J Neurosci Methods.

[26] White NR, Cagiano R, Moises AU, Barfield RJ (1990). Changes in mating vocalizations over the ejaculatory series in rats (*Rattus norvegicus*). J Comp Psychol.

[27] Fendt M, Schwienbacher I, Schnitzler HU (2006). Carbachol injections into the nucleus accumbens induce 50 kHz calls in rats. Neurosci Lett.

[28] Gulia KK, Patel N, Kumar VM (2015). Increased ultrasonic vocalizations and risk-taking in rat pups of sleep-deprived dams. Physiol Behav.

[29] Burke CJ, Kisko TM, Swiftwolfe H, Pellis SM, Euston DR (2017). Specific 50-kHz vocalizations are tightly linked to particular types of behavior in juvenile rats anticipating play. PLoS ONE.

[30] Sales GD (1972). Ultrasound and aggressive behaviour in rats and other small mammals. Anim Behav.

[31] Baturaite Z, Voipio H-M, Ruksenas O, Luodonpää M, Leskinen H, Apanaviciene N, Nevalainen T (2005). Comparison of and habituation to four common methods of handling and lifting of rats with cardiovascular telemetry. Scand J Lab Anim Sci.

[32] Korczynski R, Korda P (1988). Immobility reflex evoked by vertical lifting of the rat. Acta Neurobiol Exp (Wars).

[33] Brewster J, Leon M (1980). Facilitation of maternal transport by Norway rat pups. J Comp Physiol Psychol.

[34] Esposito G, Yoshida S, Ohnishi R, Tsuneoka Y, Rostagno Mdel C, Yokota S, Okabe S, Kamiya K, Hoshino M, Shimizu M, Venuti P, Kikusui T, Kato T, Kuroda KO (2013). Infant calming responses during maternal carrying in humans and mice. Curr Biol.

[35] Hori M, Shimoju R, Tokunaga R, Ohkubo M, Miyabe S, Ohnishi J, Murakami K, Kurosawa M (2013). Tickling increases dopamine release in the nucleus accumbens and 50 kHz ultrasonic vocalizations in adolescent rats. NeuroReport.

